# Elastography for the diagnosis of high-suspicion thyroid nodules based on the 2015 American Thyroid Association guidelines: a multicenter study

**DOI:** 10.1186/s12902-020-0520-y

**Published:** 2020-04-03

**Authors:** Li Hairu, Peng Yulan, Wang Yan, Ai Hong, Zhou Xiaodong, Yang Lichun, Yan Kun, Xiao Ying, Liu Lisha, Luo Baoming, Yong Qiang, Cong Shuzhen, Jiang Shuangquan, Fu Xin, Ma Buyun, Li Yi, Zhang Xixi, Gong Xue, Chen Haitao, Liu Wenying, Tang Ling, Lv Xiaoyu, Zhao Xinbao, Li Liang, Gan Kehong, Tian Jiawei

**Affiliations:** 1 Department of Ultrasound, The Second Affiliated Hospital of Harbin Medical University, Harbin, 150086 Heilongjiang Province China; 2 Department of Diagnostic Ultrasound and The National Key Discipline of Medical Imaging and Nuclear Medicine, West China Hospital of Sichuan University, Chengdu, Sichuan Province China; 3 Department of Ultrasound, Sixth People’s Hospital Affiliated to Shanghai Communication University, Shanghai, China; 4 Department of Ultrasound, First Affiliated Hospital of Xi’an Communication University, Xi’an, Shanxi Province China; 5 Department of Ultrasound, Xijing Hospital Affiliated to The Fourth Military Medical University, Xi’an, Shanxi Province China; 6 Department of Ultrasound, Third Affiliated hospital of Kunming Medical University, Kunming, Yunnan Province China; 7 Department of Ultrasound, Tumor Hospital of Beijing University, Beijing, China; 8 Department of Ultrasound, Xiang-ya Hospital of Centre-south University, Changsha, Hunan Province China; 9 Department of Ultrasound, Tumor Hospital Affiliated to Xinjiang Medical University, Urumqi, Xinjiang Province China; 10 Department of Ultrasound, Sun Yat-sen Memorial Hospital of Sun Yat-sen University, Guangzhou, Guangdong Province China; 11 Department of Ultrasound, Beijing Anzhen Hospital Affiliated to Capital Medical University, Beijing, China; 12 Department of Ultrasound, People’s Hospital of Guangdong Province, 106 Zhongshan Second Road, Guangzhou, Guangdong China

**Keywords:** Elastography, Thyroid, Cancer, Ultrasound

## Abstract

**Background:**

An accurate diagnosis for high-suspicion nodules based on the 2015 American Thyroid Association (ATA) guidelines would reduce unnecessary invasive examinations. Elastography is a useful tool for discriminating benign and malignant thyroid nodules. The aim of this study is to investigate the diagnostic efficiency of elastography for high-suspicion thyroid nodules based on the 2015 ATA guidelines in the Chinese population.

**Methods:**

Thyroid nodules with high-suspicion characteristics based on the 2015 ATA guidelines were subjected to conventional ultrasound (US) and ultrasound strain elastography (USE) examinations at 12 hospitals from 4 geographic regions across China. Cytology/histology of thyroid nodules was used as a reference method. Receiver operating characteristic (ROC) curves were plotted to evaluate the diagnostic performance of the elasticity score (ES) and strain ratio (SR). Logistic regression analysis was used to determine the predictors of malignancy.

**Results:**

Overall, a total of 1445 thyroid nodules (834 malignant, 611 benign) from 12 centers were included in the final analysis. The areas under the curve of the ES and SR were 0.828 and 0.732, respectively. The sensitivity, specificity, accuracy, positive predictive value (PPV) and negative predictive value (NPV) of the ES were 92.4, 60.7, 79.0, 76.3 and 85.5%, respectively, and those of the SR were 81.1, 50.1, 68.9, 65.9 and 67.9%, respectively. The combination of the Thyroid Imaging Reporting and Data System (TI-RADS) and ES led to a significant increase in the sensitivity and NPV (97.1 and 91.9%, respectively) compared with the TI-RADS alone. Logistic regression analysis showed that microcalcifications (OR = 5.290), taller than wide (OR = 12.710), irregular margins (OR = 10.117), extrathyroidal extension (ETE; OR = 6.412), the ES (OR = 3.741) and the SR (OR = 1.083) were independent predictors of malignant thyroid nodules. The sensitivity, specificity, accuracy, PPV and NPV of the ES were all superior in nodules ≥1 cm than in those < 1 cm (95.0% vs 90.4, 68.8% vs 56.8, 85.9% vs 74.4, 85.2% vs 69.9, and 87.8% vs 84.2%, respectively).

**Conclusions:**

Elastography combined with the ES is a valuable tool for the assessment of high-suspicion thyroid nodules based on the 2015 ATA guidelines, especially in nodules ≥1 cm.

## Background

Thyroid nodules are common diseases, and their incidence has increased rapidly during the past decades. Thyroid nodules detected by ultrasound (US) have been detected in up to 50% of the general population [1]. Although most nodules are benign, asymptomatic and do not require treatment, a reliable diagnosis is still necessary to achieve the optimal therapeutic schedule [2]. The early detection and differentiation of malignant thyroid nodules from overall thyroid nodules are particularly important for planning treatment and evaluating prognosis. A diagnostic thyroid US examination is recommended for all patients with thyroid nodules according to the American Thyroid Association (ATA) guidelines [3]. Although certain sonographic features, such as taller than wide, blurred margins, irregular borders, internal microcalcifications, hypoechogenicity and marked hypoechogenicity [4], are conventional indicators of malignancy, the sensitivity and specificity vary largely from 38.8 to 90.9% and 53.0 to 96.6%, respectively [5–7].

Ultrasound strain elastography (USE) has been proposed for differentiating malignant thyroid nodules from benign nodules based on their elasticity and has gradually developed into a widely used US examination method [8]. USE produces an elasticity score (ES) as the qualitative output and the strain ratio (SR, the ratio obtained by dividing the mean strain within the lesion by the mean strain of the surrounding normal tissue) as a semiquantitative output in units of relative strain. USE is based on the principle that softer parts deform more easily than harder parts when body tissues are compressed. Studies have shown that USE can differentiate malignant thyroid nodules from benign nodules with high sensitivity and specificity [9, 10]. However, several studies have found contradictory results in which USE had a lower sensitivity and specificity than conventional US [11, 12]. Thus, the efficacy of USE remains controversial, and additional studies are needed to confirm its efficacy.

According to the 2015 ATA guidelines, the malignant risk of high-suspicion thyroid nodules is > 70–90% [3]. However, the ATA characteristics of high-suspicion thyroid nodules overlap with degrees 4a to 5 of Kwak’s Thyroid Imaging Reporting and Data System (TI-RADS), the malignancy of which ranges from 3.3 to 87.5% [13]. Depending on the discrepancy between the two methods, we performed a nationwide, multicenter study in China to ascertain the efficacy of USE in differentiating benign and malignant high-suspicion thyroid nodules based on the 2015 ATA guidelines. To our knowledge, this is the first multicenter study in China to investigate the diagnostic performance of strain elastography in differentiating malignant from benign thyroid nodules of high suspicion based on the 2015 ATA guidelines in a large population.

## Methods

### Patients

**Fig. 1:**
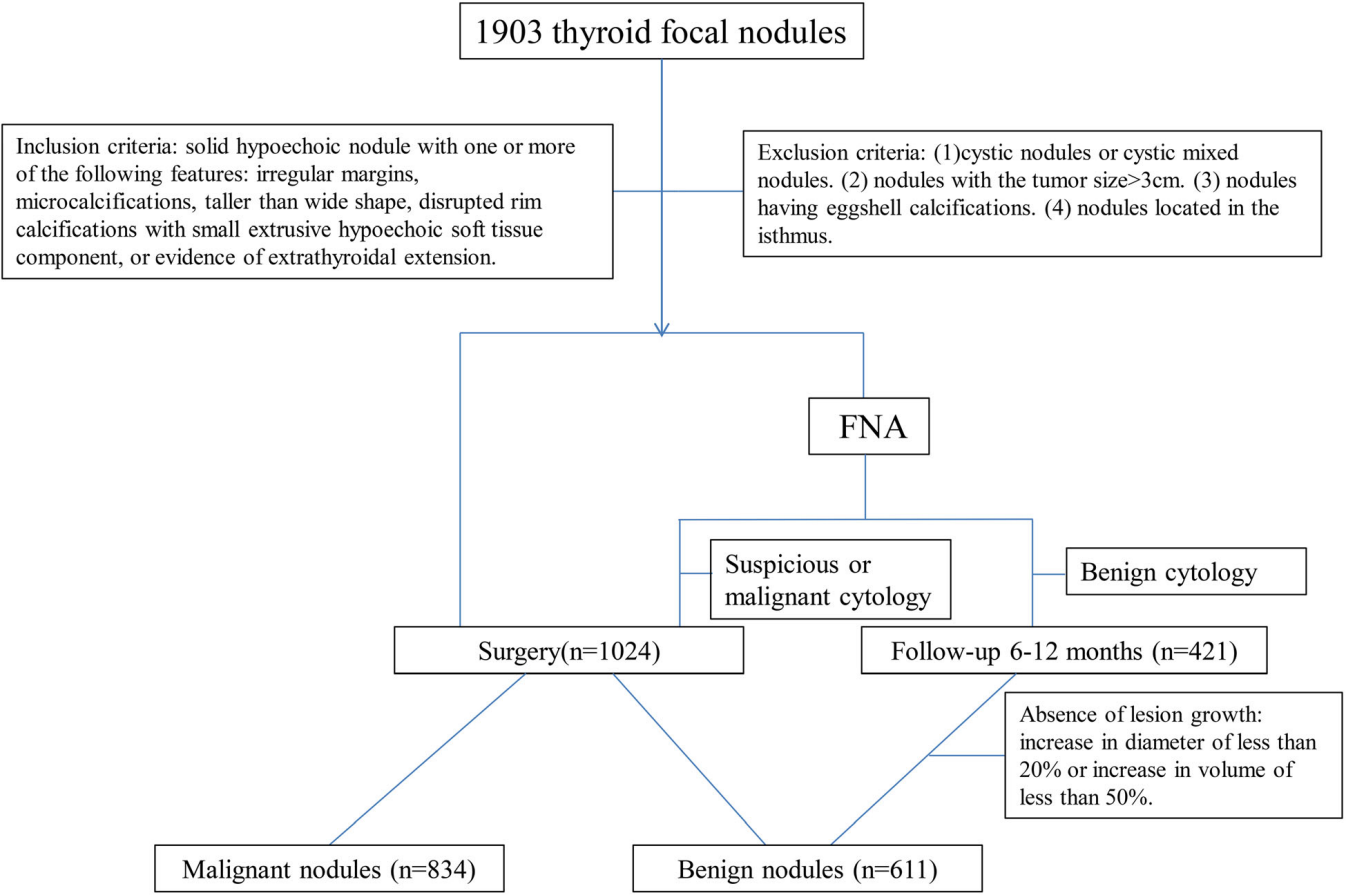
Nodule selection among the included patients

Between March 2014 and September 2018, 1819 patients with 1903 suspicious nodules at 12 diagnostic centers from 4 geographic regions across China were evaluated by conventional US and USE (Fig. 1). The inclusion criteria were solid hypoechoic nodules with at least one of the following features: irregular margins, microcalcifications, taller than wide, extrusive hypoechoic soft tissue surrounded disrupted rim calcifications, and extrathyroidal extension (ETE) [3]. The exclusion criteria were as follows: cystic nodules or cystic mixed nodules, nodules with a tumor size > 3 cm, nodules with eggshell calcifications, and nodules located in the isthmus. In total, 1445 thyroid nodules were classified as high-suspicion nodules and included in the final analysis. The male/female ratio was 304/1141. The mean age of the patients with benign nodules was 48.30 ± 10.86 years, and the mean age of the patients with malignant nodules was 42.49 ± 11.39 years. The ethics committee of each hospital approved this study. Written informed consent for participation was obtained from all patients.

### Equipment

All studies were performed using the same type of US machine, i.e., HI VISION Ascendus, HI VISION Avius or HI VISION PREIRUS (Hitachi Medical, Tokyo, Japan), which had both conventional B-mode US and elastography capabilities. A 5–13 MHz linear transducer probe was used for conventional US and USE examinations.

### US and USE examinations

Both conventional US and real-time USE were conducted and recorded by two skilled sonographers at each center with at least 5 years of conventional US experience and 1 year of USE experience. Final results were identified by two examiners. If the results of the two examiners were inconsistent, then a superior physician was invited to make a final decision. All examiners were blinded to the results of the pathological diagnosis. The examination method, procedure, and conditions used by the inspectors remained consistent for all patients. For the elastography examination, the USE/US double-image display mode was selected. Once the default thyroid conditions were selected, they were not changed throughout the process of the USE examination. Next, the region of interest (ROI) of USE was selected as follows: the lesion area was no more than 1/3 of the ROI area to ensure that sufficient surrounding normal tissue was available as a reference. The ROI included a small amount of anterior cervical muscle tissue, the thyroid gland, and the thyroid gland capsule, and cervical blood vessels and tracheal tissue were avoided to the greatest extent possible. The ROI was not set within 2 mm of the skin surface.

**Fig. 2:**
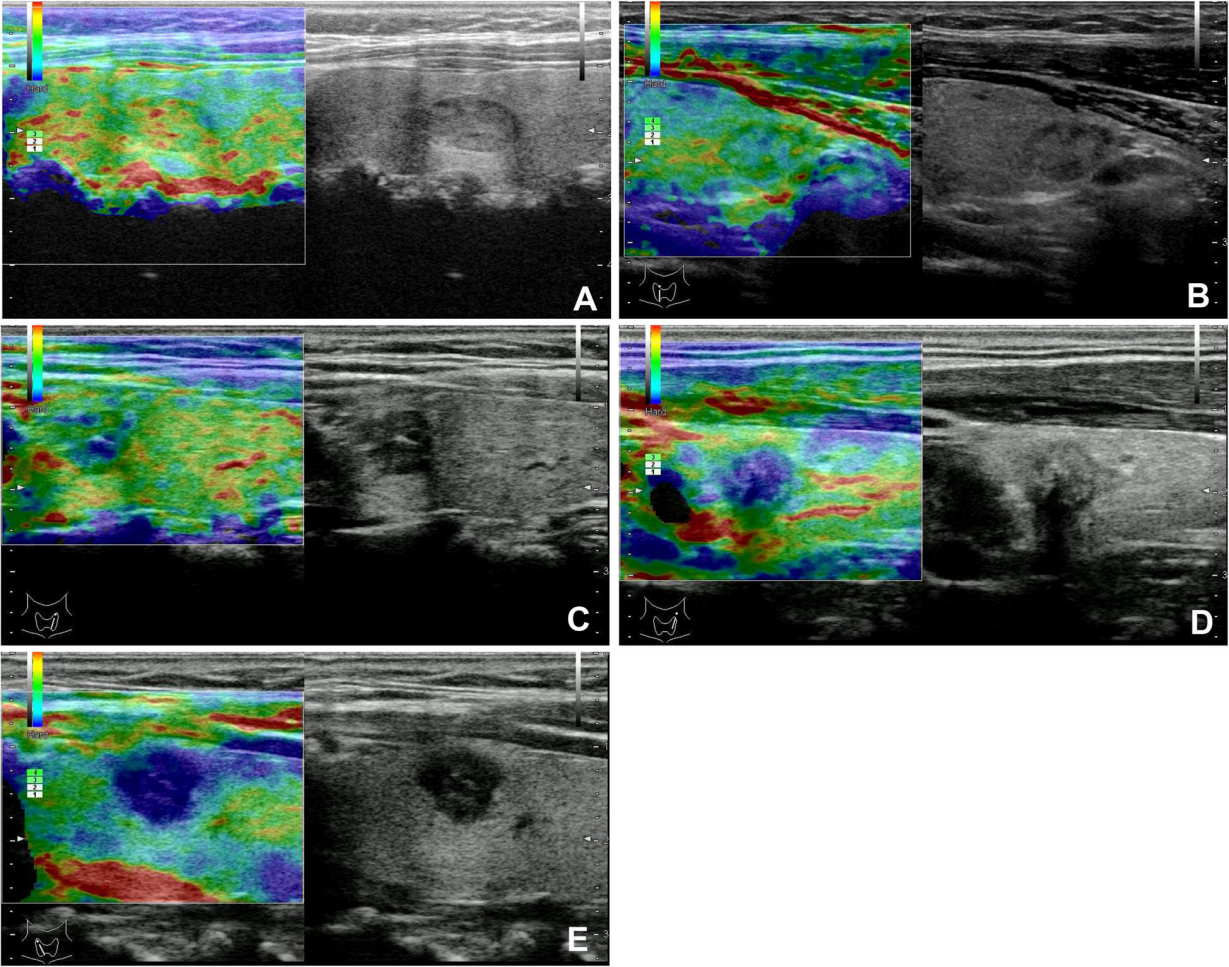
US characteristics of different ESs. **a** USE image of an adenoma in a 45-year-old man. On the left side, the nodule shaded in green and red was scored as 1. **b** USE image of a nodular goiter in a 45-year-old woman. On the left side, the nodule shaded in green with focal blue s*p*ots was scored as 2. **c** USE image of a tiny papillary thyroid microcarcinoma in a 34-year-old woman. On the left side, the nodule shaded half in green and half in blue was scored as 3. **d** USE image of a tiny papillary thyroid microcarcinoma in a 39-year-old man. On the left side, the nodule shaded in blue with focal green spots was scored as 4. **e** USE image of a papillary thyroid microcarcinoma in a 60-year-old woman. On the left side, the entire nodule and its surrounding area in blue were scored as 5

According to Itoh et al. [14], the elastograms of the thyroid lesions were classified into 5 types. Even strain of the entire hypoechoic lesion was indicated as score 1 (Fig. 2a). Strain in most of the hypoechoic lesion, with certain areas of no strain was indicated as score 2 (Fig. 2b). Strain at the periphery of the hypoechoic lesion and no strain at the center of the lesion was indicated as score 3(Fig. 2c). No strain at the entire hypoechoic lesion was indicated as score 4(Fig. 2d). No strain at the entire hypoechoic lesion and surrounding area was indicated as score 5(Fig. 2e).

SR measurement: The operator traced the outline of the lesion in the two-dimensional figure or the elastic image on the longitudinal views. Then, the area adjacent to the target lesion, which was at the same depth as the lesion, was selected as a reference. The SR was automatically calculated by the software.

### Reference standard

Fine-needle aspiration (FNA) was performed as recommended by the 2015 ATA guidelines [3]. Patients with suspicious or malignant cytology were referred for surgery. Some of the patients who underwent thyroidectomy did not receive FNA due to certain reasons, such as highly suspicious malignancy or refused FNA. The patients who underwent surgery were subjected to a pathological section examination, and the pathology results were used as the gold standard. Benign nodules confirmed by FNA were followed up for 6–12 months and without lesion growth (less than 20% increase in diameter). Two experienced pathologists at each center with at least 5 years of working experience blinded to the US and USE results performed the cytology and histology.

### Statistical analysis

The measurement data are expressed as the mean ± standard deviation. A t test was used to analyze measurement. The counting data are presented as cases (percentages), and analyzed by chi-square test. Logistic regression analysis was used for risk factor analysis of both benign and malignant nodules. ROC curves and AUC values were used to examine the diagnostic efficiency of each index. A *p* < 0.05 on both sides was considered statistically significant.

## Results

### Clinicopathologic data

A total of 1819 patients with 1903 nodules were enrolled from 12 centers in this multicenter study. One hundred and twenty-six patients were excluded due to the absence of adequate data on USE, cytology or histology results or were lost to follow-up, and 332 nodules were not high-suspicion nodules. Therefore, the final analysis comprised 1445 thyroid nodules, including 834 malignant nodules and 611 benign nodules. All 834 malignant nodules and 190 benign nodules were confirmed by surgery, and the remaining 421 benign nodules were confirmed by a cytologic examination. Of the 834 malignant nodules, 690 were papillary carcinomas, 133 were papillary microcarcinomas, 7 were follicular carcinomas, and 4 were medullary carcinomas. The final diagnosis of benign histology was adenoma in 38 nodules, nodular goiter in 121 nodules, follicular nodule in 9 nodules, chronic lymphocytic thyroiditis in 13 nodules, subacute granulomatous thyroiditis in 3 nodules, hyperplasia of the thyroid tissue in 1 nodule, inflammatory lesion in 4 nodules and tuberculosis in 1 nodule.

### Diagnostic performance of conventional US

**Fig. 3:**
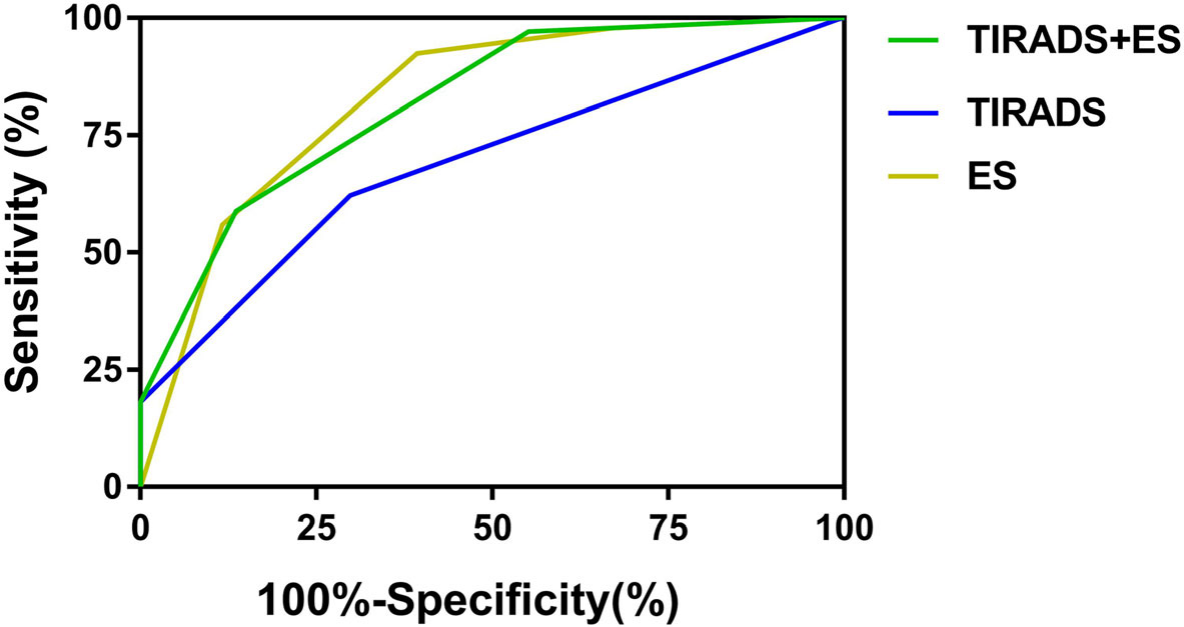
ROC curve of the TI-RADS, ES and TI-RADS combined with ES in differentiating malignant and benign nodules

The results of the diagnostic performance of conventional US and USE are shown in Table 1. All of the parameters were significantly different between the two groups (*p < 0.005*). The sensitivity, specificity, accuracy, positive predictive value (PPV) and negative predictive value (NPV) were calculated for each suspicious criterion. Irregular margins and ETE had a high sensitivity (93.9 and 83.0%, respectively) but very low specificity (14.1 and 23.2%, respectively). Taller than wide and disrupted rim calcifications with a small extrusive hypoechoic soft tissue component had a high specificity (91.0 and 98.9%, respectively) but low sensitivity (37.1 and 4.7%, respectively). The sensitivity and specificity of microcalcifications were moderate (49.9 and 65.6%, respectively). As Kwak’s TI-RADS is the mainstream classification of malignant degree, we analyzed the diagnostic performance of Kwak’s TI-RADS. Kwak’s TI-RADS score showed a sensitivity of 62.2%, a specificity of 70.2%, a PPV of 74.0%, an NPV of 57.7% and an AUC under the ROC curve of 0.69 in the overall assessment (Table 1, Fig. 3).

**Table 1:** Diagnostic value of US and USE for the diagnostic of malignant thyroid nodules

Criteria	Benign	Malignant	Sensitivity (%)	Specificity (%)	Accuracy (%)	PPV (%)	NPV (%)	*P*
Microcalcification								
Yes	211	416	49.9	65.6	56.4	66.3	48.9	<0.01
no	400	318						
Taller than wide								
yes	55	309	37.1	91.0	59.8	84.9	51.4	<0.01
no	556	525						
Irregular margins								
Yes	525	83	93.9	14.1	60.1	59.9	62.8	<0.01
no	86	51						
ETE								
Yes	469	693	83.0	23.2	57.7	59.6	50.0	<0.01
No	142	141						
Disrupted rim calcification								
Yes	7	39	4.7	98.9	44.4	84.8	43.2	<0.01
No	604	795						
Kwak’s TI-RADS								
4a	5	2	62.2	70.2	65.6	74.0	57.7	<0.01
4b	424	313						
4c	182	367						
5	0	152						
Elasticity score								
> 3	240	771	92.4	60.7	79.0	76.3	85.5	<0.01
≤ 3	371	63						
SR value								
> 2.99	305	676	81.1	50.1	68.9	65.9	67.9	<0.01
≤ 2.99	306	158						

*PPV* positive predictive value, *NPV* negative predictive value, *ETE* Extrathyroidal extension, *TI-RADS* Thyroid Imaging Reporting and Data System, *SR* strain ratio

### Diagnostic performance of USE

There were 21 nodules with an ES of 1 (20 benign, 1 malignant), 198 nodules with an ES of 2 (181 benign, 17 malignant), 215 nodules with an ES of 3 (170 benign, 45 malignant), 473 nodules with an ES of 4 (169 benign, 304 malignant), and 538 nodules with an ES of 5 (71 benign, 467 malignant). There were significant differences in the ES and SR values between the malignant and benign groups (*p* < 0.001). The malignant percentage increased with the increase in ES, with values of 4.8, 8.6, 20.9, 64.3 and 86.8%, respectively.

**Fig. 4:**
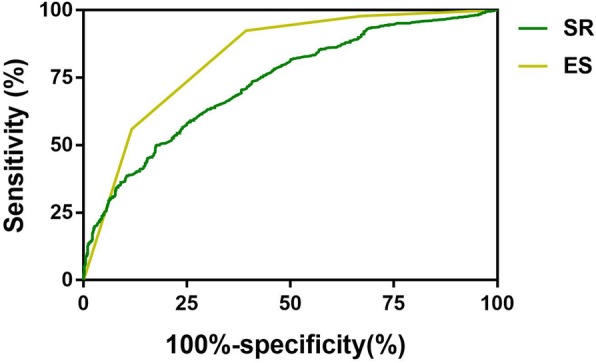
ROC curve of the ES and SR value in differentiating malignant and benign nodules

The mean (±SD) ES of the malignant and benign nodules was 3.15 ± 1.072 and 4.46 ± 0.703, respectively (*p* < 0.001). Figure 4 shows the ROC curve of the ES and SR in differentiating malignant and benign nodules. The AUC of the ES was 0.828. The optimal cut-off value of the ES was 3.5 in this study. The sensitivity, specificity, accuracy, PPV and NPV of the ES were 92.4, 60.7, 79.0, 76.3 and 85.5%, respectively. The mean (±SD) SR of the malignant and benign nodules was 3.26 ± 2.06 and 6.15 ± 7.21, respectively (*p* < 0.001). The AUC of the SR was 0.732. The optimal cut-off value of the SR was 2.99. The sensitivity, specificity, accuracy, PPV and NPV of the SR were 81.1, 50.1, 67.9, 68.9 and 65.9%, respectively (Table 1). The combination of the TI-RADS with ES led to a significant increase in the sensitivity, NPV (97.1 and 91.9%, respectively) and the AUC under the ROC curve (0.819) (Fig. 3).

### Logistic regression analysis for predicting malignant thyroid nodules

In the univariate and multivariate logistic regression analyses, microcalcification (OR = 1.887 and 5.29, *p* < 0.001, respectively), taller than wide (OR = 5.950 and 12.71, *p* < 0.001, respectively), irregular margins (OR = 2.515 and 10.117, *p* < 0.001, respectively), the ES (OR = 4.473 and 3.741, *p* < 0.01, respectively) and the SR (OR = 1.504 and 1.083, *p* < 0.01, respectively) were statistically significant predictors of malignant thyroid nodules (Table 2). ETE was a predictor of malignant nodules in the univariate analysis but not in the multivariate analysis (*p* > 0.05).

**Table 2:** Logistic regression analysis for predicting malignant thyroid nodules

Criteria	Univariate analysis			Multivariate analysis		
	OR	95%CI	*P*	OR	95%CI	*P*
Microcalcification	1.887	1.522–2.339	0.000	5.29	3.472–8.078	0.001
Taller than wide	5.950	4.361–8.118	0.000	12.71	7.625–21.188	0.000
Irregular margins	2.515	1.748–3.618	0.000	10.117	5.88–17.404	0.000
Rim calcification	4.233	1.880–9.529	0.000	2.569	0.791–8.348	0.117
ETE	1.475	1.137	1.915	6.412	3.909–10.519	0.000
Elasticity score	4.473	3.824–5.231	0.000	3.741	3.129–4.474	0.000
SR value	1.504	1.405–1.610	0.000	1.083	1.028–1.142	0.003

*SR* strain ratio, *OR* odds ratio, *CI* confidence interval

### Diagnostic performance of the ES and SR in nodules of different sizes

According to the 2015 ATA thyroid nodule guidelines, nodules ≥1 cm in the largest dimension with a high-suspicion sonographic pattern are recommended for FNA; thus, we compared the diagnostic performance of the ES and SR in nodules ≥1 cm and < 1 cm. There were 877 nodules < 1 cm and 568 nodules ≥1 cm. The male/female ratios were 180/697 and 124/444, respectively, and the mean age of the patients was 45.58 ± 11.001 years for those with nodules < 1 cm and 44.01 ± 12.268 years for those with nodules ≥1 cm. The results presented in Table 3 demonstrate that the AUC, sensitivity, specificity, accuracy, PPV and NPV of the ES were all superior in nodules ≥1 cm than in those < 1 cm (95.0% vs 90.4, 68.8% vs 56.8, 85.9% vs 74.4, 85.2% vs 69.9, and 87.8% vs 84.2%, respectively), with especially high accuracy. The specificity, accuracy and PPV of the SR were also higher in nodules ≥1 cm than in those < 1 cm (59.3% vs 45.6, 72.7% vs 64.8, and 78.8% vs 62.6%, respectively). The above data indicate that elastography with ES has an excellent diagnostic performance in nodules ≥1 cm.

**Table 3:** Diagnostic performance of Elasticity score and SR in different size of nodules

	AUC	Sensitivity	Specificity	Accuracy	PPV	NPV	P
Elasticity score							
≥ 1	0.875	95.0	68.8	85.9	85.2	87.8	0.000
<1	0.800	90.4	56.8	74.4	69.9	84.2	0.000
SR							
≥ 1	0.779	79.8	59.3	72.7	78.8	60.8	0.000
<1	0.709	82.1	45.6	64.8	62.6	69.6	0.000

*PPV* positive predictive value, *NPV* negative predictive value, *SR* strain ratio

## Discussion

With the development of high-frequency ultrasonic devices, more thyroid nodules have been diagnosed. Although most thyroid nodules are benign, a diagnosis differentiating malignant from benign nodules is still of vital importance for determining the appropriate therapy. A differential diagnosis is still a difficult problem for clinicians. Elastography has emerged as a valuable technology to assist in the diagnosis of thyroid nodules due to its ability to reflect the stiffness of tissue that is related to malignant lesions. A large number of studies have shown the excellent performance of elastography in the differentiation of malignant and benign nodules [15–17]. Nevertheless, several studies have revealed the opposite result [11, 12]. The present study was the first multicenter study in China with a large sample size to determine the value of real-time strain elastography in the discrimination of malignant from benign thyroid nodules with high-suspicion characteristics based on the 2015 ATA guidelines. The results showed that the combination of USE with the ES had good efficacy for the discrimination of malignant from benign high-suspicion thyroid nodules, with a high sensitivity (92.4%), accuracy (79.0%) and NPV (85.5%), especially in nodules ≥1 cm (95.0, 85.9, and 87.8%, respectively). Additionally, logistic regression analysis showed that the ES and SR were independent predictors of malignancy.

US is the preferred technology for thyroid nodules due to its convenience, maneuverability and low cost. Conventional US malignant characteristics, including microcalcifications, hypoechogenicity, irregular margins, and taller than wide were significantly different between malignant and benign nodules in this study. Logistic regression analysis showed that microcalcifications, taller than wide, irregular margins and ETE were independent predictors of malignancy. However, previous studies have reported largely varied sensitivities and specificities [5–7]. The diagnostic efficacy of conventional US parameters in this study was also unsatisfactory. Although irregular margins and ETE yielded good sensitivity, their specificity was rather poor. Similarly, despite their high specificity, the sensitivity of taller than wide and disrupted rim calcifications with a small extrusive hypoechoic soft tissue component was unacceptable. The accuracy of conventional US parameters was also limited.

Elastography takes advantage of the change in elasticity of soft tissues resulting from specific pathological or physiological processes, and the low elasticity observed on USE is highly correlated with malignancy. Since the emergence of elastography, it has shown an outstanding sensitivity, specificity, PPV and NPV [16, 18–20], with the specificity and PPV reaching up to 100%. Elasticity, which is absent in conventional US, is the single feature with the best diagnostic performance [7], as well as a potent predictor of malignant thyroid nodules [21, 22]. With an increase in the ES, the percentage of malignancy increased significantly in this research, confirming that the ES is correlated with malignancy. In this research, we used the 5 scoring parameters proposed by Itoh, who defined an ES > 3.5 as the cut-off value; the sensitivity, specificity, accuracy, PPV and NPV were 92.4, 60.7, 79.0, 76.3 and 85.5%, respectively, with an AUC of 0.828. However, contrary opinions have also been presented. A multicenter study by Moon et al. reported that elastography showed an inferior performance in the differentiation of malignant and benign thyroid nodules relative to grayscale US features [11]. They investigated all solid nodules, including hyperechogenic, isoechogenic, hypoechogenic and marked hypoechogenic nodules, in their research. In this study, we recruited solid hypoechoic nodules according only to the recommendations of the 2015 ATA guidelines. Another study with three scoring methods showed that USE had a limited sensitivity and PPV in detecting malignant thyroid nodules and was not superior to conventional US [12]. A retrospective study involving 197 thyroid nodules utilized the iU22 system for elastography and classified the color mapping as blue versus not blue, showing that the ES and SR had a limited ability in differentiating benign from malignant thyroid nodules [23]. However, Magri et al. showed that the strain index was significantly higher in malignant thyroid nodules than in benign thyroid nodules and displayed a good diagnostic performance [24]. It can be speculated that the instruments and scoring method used are among the factors that affect the diagnostic efficacy of elastography.

Elasticity is a qualitative method; thus, it is inevitably affected by the practitioner. The SR, which is an objective and semiquantitative technology, was introduced to address this disadvantage [25]. However, whether the ES or SR is more accurate is a long-standing controversy [20, 26–28]. Furthermore, the cut-off value of SR varied largely in different studies [28, 29]. In this research, using an SR > 2.99 as the cut-off value, the sensitivity, specificity, accuracy, PPV and NPV were 81.1, 50.1, 68.9, 65.9 and 67.9%, respectively, with an AUC of 0.738, showing no advantage over the ES. Though the SR is a semiquantitative method, it is defined as the ratio obtained by dividing the mean strain within the lesion by the mean strain of the surrounding normal tissue; thus, thyroid echogenicity surrounding thyroid nodules will inevitably affect the SR. As a result, it might be inferred that no additional information is provided by the SR, which is more time consuming than the ES, in the discrimination of malignant from benign thyroid nodules. Although shear wave elastography (SWE) has drawn more attention in recent years and has been reported to be more accurate than USE in some studies [30–32], Tian et al’s and Hu et al’s meta-analysis showed that USE had a better diagnostic performance than SWE, with comparable specificity between methods [33, 34]. Even more recently, a prospective study comprising 243 nodules revealed that USE yielded the highest performance compared with the TI-RADS score and SWE [35]. The aforementioned data confirm that USE is still a promising diagnostic tool for discriminating malignant from benign thyroid nodules.

Another controversial topic is whether the combined application of US and ES may provide better results for thyroid nodule characterization. The results of the present research demonstrated that the sensitivity and NPV were dramatically higher with the combination than with the TI-RADS and ES alone, and the accuracy was higher with the combination than with the TI-RADS alone but not with the ES alone, consistent with the study by Cantisani [35]. However, the specificity decreased after the two were combined.

According to the 2015 ATA guidelines, nodules ≥1 cm in the largest dimension with a high-suspicion sonographic pattern are recommended for FNA. We compared the diagnostic efficacy of elastography in nodules ≥1 cm and < 1 cm, and the results showed that the AUC of the ES for nodules ≥1 cm was 0.875, with excellent sensitivity, accuracy, PPV, and NPV and good specificity. The aforementioned parameters were all superior to those of nodules < 1 cm. The SR demonstrated a similar trend, except it had a lower sensitivity and NPV in nodules ≥1 cm than in nodules < 1 cm. Wang’s research also showed a similar result: elastography yielded higher sensitivity for nodules larger than 1 cm [36]. It might be concluded that elasticity is a helpful tool for discriminating benign and malignant thyroid nodules. FNA is not popular in many developing countries because of the lack of skillful cytologists and the high dependence on the skill and experience of the operator and cytologist [37]. Furthermore, FNA is an invasive technique that is time consuming and costly. Thus, the use of FNA is limited in certain areas.

This study has certain limitations. Cytology without lesion growth was also accepted as a reference method for benign lesions, as suggested by international guidelines, which may have resulted in selection bias. However, including only histology would increase the number of malignant lesions [16]. Furthermore, the number of follicular carcinomas and medullary carcinomas, which was determined by morbidity, was low, consistent with previous studies [16, 38]. No reproducibility tests were performed in this study because all US diagnoses were performed by two physicians.

## Conclusions

In summary, strain elastography combined with the ES is an effective technique for the discrimination of benign and malignant nodules among high-suspicion thyroid nodules based on the 2015 ATA guidelines, especially in nodules ≥1 cm.

## Data Availability

The datasets analyzed during the current study are available from the corresponding author upon reasonable request.

## Abbreviations

ATA: American Thyroid Association
ROC: Receiver operating characteristic
ES: Elasticity score
SR: Strain ratio
PPV: Positive predictive value
NPV: Negative predictive value
US: Ultrasound
USE: Ultrasound strain elastography
TI-RADS: Thyroid Imaging Reporting and Data System
ETE: Extrathyroidal extension
ROI: Region of interest
FNA: Fine-needle aspiration
AUC: Area under the curve
